# Glucometabolic Control and Anti-Transglutaminase Antibodies at Celiac Disease Onset in Type 1 Diabetes Youth

**DOI:** 10.1210/clinem/dgaf604

**Published:** 2025-11-04

**Authors:** Francesca Di Candia, Francesco Maria Rosanio, Roberto Franceschi, Alessandro Fierro, Riccardo Bonfanti, Francesca Cardella, Valentino Cherubini, Giuseppe D’Annunzio, Barbara Felappi, Dario Iafusco, Brunella Iovane, Claudio Maffeis, Giulio Maltoni, Francesca Olivieri, Gabriele Olivieri, Barbara Piccini, Elvira Piccinno, Barbara Predieri, Ivana Rabbone, Maria Rossella Ricciardi, Giuseppina Salzano, Riccardo Schiaffini, Gianluca Tornese, Angela Zanfardino, Marco Marigliano, Riccardo Troncone, Riccardo Pertile, Luigi Greco, Renata Auricchio, Enza Mozzillo, ISPED Diabetes Study Group Collaborators, Francesco Gallo, Caterina Grosso, Carlo Ripoli, Fiorella De Berardinis, Susanna Coccoli, Valentina Tiberi, Sonia Toni, Maurizio Delvecchio, Rosanna Roppolo, Fortunato Lombardo, Stefano Passanisi, Bruno Bombaci, Alberto Casertano, Nicola Minuto, Marta Bassi, Evelina Maines, Silvia Savastio, Elena Inzaghi, Andrea Rigamonti, Giulio Frontino, Patrizia Bruzzi, Claudia Piona

**Affiliations:** Department of Translational Medical Science, Pediatric Section, University of Federico II, 80131 Naples, Italy; Department of Translational Medical Science, Pediatric Section, University of Federico II, 80131 Naples, Italy; CISMed—Centro Interdipartimentale di Scienze Mediche, University of Trento, 38122 Trento, Italy; Department of Translational Medical Science, Pediatric Section, University of Federico II, 80131 Naples, Italy; Unit of Paediatric Diabetology, Department of Pediatrics, Diabetes Research Institute, San Raffaele Scientific Institute, 20132 Milan, Italy; Department of Pediatrics, Regional Center of Pediatric Diabetes, Children's Hospital G. Di Cristina, 90134 Palermo, Italy; Department of Women's and Children's Health, G. Salesi Hospital, Azienda Ospedaliero-Universitaria Ospedali Riuniti, 60126 Ancona, Italy; Pediatric Clinic and Endocrinology Unit, IRCCS Istituto Giannina Gaslini, 16147 Genova, Italy; U.S. Paediatric Auxoendocrinology, Complex Operative Unit of Pediatrics-Paediatric Clinic, Azienda Socio Sanitaria Territoriale (ASST) Spedali Civili, 25123 Brescia, Italy; Department of Woman, Child, and General and Specialistic Surgery, Regional Center of Paediatric Diabetes, University of Campania “L. Vanvitelli”, 80138 Naples, Italy; Unit of Paediatric Diabetology, Department of Mother and Child Pediatric, University Hospital of Parma, 43126 Parma, Italy; Paediatric Diabetes and Metabolic Disorders Unit, Regional Center for Paediatric Diabetes, University City Hospital of Verona, 37126 Verona, Italy; Department of Medical and Surgical Sciences, University Hospital S.Orsola-Malpighi, 40138 Bologna, Italy; Paediatric Diabetes and Metabolic Disorders Unit, Regional Center for Paediatric Diabetes, University City Hospital of Verona, 37126 Verona, Italy; Unit of Paediatric Diabetology, Department of Pediatrics, Diabetes Research Institute, San Raffaele Scientific Institute, 20132 Milan, Italy; Diabetology Unit, Meyer University Children's Hospital, 50134 Florence, Italy; Metabolic Disorder and Diabetes Unit, “Giovanni XXIII” Children's Hospital, 70126 Bari, Italy; Department of Medical and Surgical Sciences of Mother, Children and Adults, Paediatric Unit, University of Modena and Reggio Emilia, 41124 Modena, Italy; Division of Pediatrics, Department of Health Sciences, University of Piemonte Orientale, 28100 Novara, Italy; Paediatric Diabetology Unit, Department of Pediatrics, ASL 8 Cagliari, 09121 Cagliari, Italy; Department of Human Pathology in Adult and Developmental Age “Gaetano Barresi”, University of Messina, 98125 Messina, Italy; Diabetes Unit, Bambino Gesù Children's Hospital, 00165 Rome, Italy; Department of Medical, Surgical and Health Sciences, University of Trieste, 34129 Trieste, Italy; Department of Woman, Child, and General and Specialistic Surgery, Regional Center of Paediatric Diabetes, University of Campania “L. Vanvitelli”, 80138 Naples, Italy; Paediatric Diabetes and Metabolic Disorders Unit, Regional Center for Paediatric Diabetes, University City Hospital of Verona, 37126 Verona, Italy; Department of Translational Medical Science, Pediatric Section, University of Federico II, 80131 Naples, Italy; European Laboratory for the Investigation of Food Induced Disease (ELFID), University of Federico II, 80131 Naples, Italy; Department of Clinical and Evaluative Epidemiology, Trento Health Service (APSS), 38123 Trento, Italy; Department of Translational Medical Science, Pediatric Section, University of Federico II, 80131 Naples, Italy; European Laboratory for the Investigation of Food Induced Disease (ELFID), University of Federico II, 80131 Naples, Italy; Department of Translational Medical Science, Pediatric Section, University of Federico II, 80131 Naples, Italy; European Laboratory for the Investigation of Food Induced Disease (ELFID), University of Federico II, 80131 Naples, Italy; Department of Translational Medical Science, Pediatric Section, University of Federico II, 80131 Naples, Italy; European Laboratory for the Investigation of Food Induced Disease (ELFID), University of Federico II, 80131 Naples, Italy

**Keywords:** type 1 diabetes, celiac disease, anti-tissue transglutaminase, glycosylated hemoglobin, gluten-free diet, autoimmunity

## Abstract

**Context:**

Anti-transglutaminase antibodies (anti-TTG IgA) titer is associated with mucosal damage in celiac disease (CD).

**Objective:**

The primary focus was to correlate anti-TTG IgA titer, HbA1c when CD occurs (HbA1cCD), and Marsh grade in children and adolescents with type 1 diabetes (T1D) at the time of CD diagnosis. As secondary outcomes, we assessed the optimal anti-TTG IgA upper limit of normal (ULN) cutoff for sparing biopsy, and personal and familial autoimmunity history in the individuals with T1D and CD (T1D-CD) compared with T1D-only.

**Methods:**

In this retrospective observational study, among 6933 individuals with T1D onset (2010-2019), 556 were grouped according to CD onset: before (CD_FIRST), concomitant (CD_CONCOMITANT), or after T1D (T1D_FIRST), and compared with 141 T1D without CD. Measures included HbA1cCD, fold-anti-TTG IgA, anti-TTG IgA cutoff, and autoimmunity history of both groups, as well as Marsh grade in T1D-CD.

**Results:**

In youths with T1D, HbA1cCD was associated with increased fold-anti-TTG IgA (Spearman *r* = 0.14, *P* = .0047). The optimal anti-TTG IgA cutoff for sparing biopsy was 11 ULN. Autoimmunity was prevalent in T1D-CD individuals, who showed more comorbidities than controls (χ^2^ 25.4, *P* < .001), particularly the CD_FIRST (*P* < .001).

**Conclusion:**

In children with T1D-CD, worse glucometabolic control is associated with an increase in fold anti-TTG IgA and with worse Marsh grade. A slightly higher anti-TTG IgA cutoff may be necessary for sparing biopsy compared to children in the general population. Higher prevalence of autoimmune comorbidities in CD_FIRST suggests that screening for T1D in the CD population should be mandatory.

Children and adolescents with type 1 diabetes (T1D) have always been considered at risk for celiac disease (CD) (1). The CD prevalence varies from 0.7% (based on biopsy results) to 3.5% (based on serological test results) in the general population (2), whereas in T1D and CD (T1D-CD) pediatric individuals, it is about 1.6% to 16.4% (3-6). Data on 4322 T1D-CD Italian children and adolescents showed a prevalence of 6.8% (7). CD can occur before (CD_FIRST), concomitantly (CD_CONCOMITANT), and after T1D onset (T1D_FIRST) (8). The International Society for Pediatric and Adolescent Diabetes (ISPAD) recommends monitoring youths with T1D for CD with anti-transglutaminase IgA antibodies (anti-TTG IgA) screening at the T1D onset and every 1 to 2 years thereafter, or more frequently if CD symptoms occur, or if there is a positive story of first-degree relative with CD (9). However, a survey among ISPAD pediatric diabetes specialists found that this management is not always adopted (10). Since 2020, the European Society of Pediatric Gastroenterology Hepatology and Nutrition (ESPGHAN) has updated criteria for CD diagnosis (11). A biopsy-free approach was established for the diagnosis of CD in the general pediatric population, in individuals with anti-TTG IgA ≥10 times the cutoff, with confirmation of anti-endomysial IgA antibody positivity in a second sample, even in asymptomatic individuals. For individuals with T1D, in a small cohort of children and adolescents, the optimal anti-TTG IgA cutoff for the diagnosis of CD avoiding biopsy was similar: 11 times the upper limit of normal (ULN) (sensitivity 87% and specificity 73%) (12). In CD, there is clear evidence of a correlation between anti-TTG IgA and mucosal lesions at the time of diagnosis (13), and in the case of nonadherence to a gluten-free diet (GFD) during follow-up (14).

In literature, several studies highlight the association between anti-TTG IgA, glucometabolic control, and adherence to the GFD in individuals with T1D and CD (15-18). However, whether there is also an association with intestinal mucosal damage has never been studied. Furthermore, there are no data on the association between anti-TTG IgA, mucosal damage, and glucometabolic control at the diagnosis of CD.

The primary outcome of this retrospective study was to evaluate, in a large cohort of children and adolescents with T1D-CD, the correlation between glucometabolic control at CD diagnosis (HbA1cCD), with the anti-TTG IgA level, and intestinal mucosal damage grade. The secondary outcomes were to assess the optimal and diagnostic cutoff of anti-TTG IgA in a large cohort with double autoimmunity (T1D-CD), and the possible association between family history and personal history of autoimmune comorbidities in youths with T1D-CD compared to a T1D-only group.

## Methods

### Patients Enrolled

This retrospective observational study was conducted by the Diabetes Study Group of the Italian Society of Paediatric Endocrinology and Diabetology (ISPED). The study, named CELDIA10-19, involved 23 Italian tertiary and secondary pediatric diabetes centers, including 11 in the North, 3 in the Center, and 9 in the South of Italy (Verona, Milan, Trento, Novara, Brescia, Trieste, Genoa, Alessandria, Bologna, Modena, Parma, Florence, Ancona, Rome, Francavilla Fontana, Naples Federico II, Naples Campania-Vanvitelli, Bari, Brindisi, Cosenza, Messina, Palermo, and Cagliari). The study was conducted following the Declaration of Helsinki and adhered to good clinical practice guidelines for research involving human individuals, approved by the Ethics Committee of the coordinating center, University of Naples “Federico II” (protocol number 380/19), as well as by the Ethics Committees of the participating centers.

We enrolled T1D-CD individuals with T1D onset between January 2010 and December 2019. Inclusion criteria were age between 0 and 20 years; availability of clinical, laboratory, and histological data; diagnosis and follow-up of T1D and CD according to current guidelines. The exclusion criteria were as follows: non-autoimmune diabetes; age > 20 years; IgA deficiency; clinical, laboratory, and histological data not available; GFD started before a diagnosis of CD; potential CD; diagnosis of CD not made according to current ESPGHAN guidelines; absence of annual screening for CD. A comparable group of T1D-only individuals, matched for sex, age, and disease duration, was concomitantly enrolled at the coordinating center.

Based on current guidelines, T1D diagnosis was confirmed if at least one autoantibody was positive (19). The diagnosis of CD was based on the following ESPGHAN guidelines (esophagogastroduodenoscopy with compatible duodenal biopsies in the case of elevated anti-TTG IgA values or without endoscopy in the case of symptomatic patients with anti-TTG IgA values >10 times ULN, endomysium-positive antibodies, and predisposing HLA) (20). When needed, according to ESPGHAN guidelines, the diagnosis of CD was confirmed by biopsies demonstrating the presence of diagnostic histology (Marsh grade 2, 3a, 3b, or 3c) alongside positive celiac serology. During follow-up of these patients, serological tests for anti-TTG IgA antibodies were routinely conducted, according to international guidelines (9): if a positive serology for CD was found without any histological changes on duodenal biopsy, this was considered a potential case of CD (20). These individuals have been excluded from this study.

### Laboratory Measurements of Autoantibodies

Anti-glutamic acid decarboxylase antibody presence (anti-GAD) was analyzed using Medipan. Medipan for anti-GAD, referring to the Medizym® anti-GAD M assay by Medipan GmbH, was a quantitative immunoassay that detects autoantibodies against glutamic acid decarboxylase (GAD65) in human serum (RRID: AB_3094514). The reference range for anti-GAD was ≥ 5 U/mL. Anti-insulinoma-associated protein 2 antibody (anti-IA2) was analyzed using Medizym® anti-IA2 M, Medipan & GA Generic Assays. The Medizym® anti-IA2 M for IA2 autoantibody was a quantitative enzyme immunoassay that measures autoantibodies to protein tyrosine phosphatase in human serum, serving as an aid in the diagnosis of T1D (RRID: AB_2889854). The reference range for anti-IA2 was ≥ 10 U/mL. Insulin autoantibodies (IAA) were measured using a quantitative enzyme-linked immunosorbent assay (ELISA) for determining IgG antibodies against insulin in human serum (RRID: AB_11186602). The reference range for anti-IAA was ≥ 0.4 U/mL. The anti–zinc transporter 8 (anti-ZnT8) was analyzed using RSR-ZnT8 Ab ELISA (bridging). The RSR-ZnT8 Ab ELISA (bridging) was a test kit used to quantitatively measure zinc transporter 8 autoantibodies (ZnT8 Ab) in human serum, serving as a serological marker to aid in the diagnosis and prediction of T1D. The “bridging” principle allowed the ZnT8 autoantibodies to bind both to ZnT8 coated on the plate and to liquid ZnT8-biotin, enabling the detection of these autoantibodies (RRID: AB_3065011). The reference range for ZnT8 Ab was ≥15 U/mL.

Anti-TTG IgA was analyzed using different anti-TTG assay methods. The Eu-tTG (EIA) for Anti-TTG IgA from Eurospital was an ELISA (RRID: AB_3717565). The reference range was >16 U/mL. The EliA™ Celikey for Anti-TTG IgA and Anti-TTG IgG was a fluorescence enzyme-linked immunoassay (FEIA). Running on the automated Phadia system, these assays used recombinant human TTG as the antigen to identify autoimmune markers associated with CD (RRID: AB_3675960). The reference range was >10 U/mL. The DiaSorin LIAISON® Anti-TTG IgA was a fully automated chemiluminescence immunoassay (CLIA) used for the quantitative determination of tissue transglutaminase autoantibodies of the IgA class in human serum or plasma. The assay ran on DiaSorin's automated LIAISON® system, designed for high-throughput laboratories (RRID: AB_3717564). The reference range was >10 U/mL.

### Study Subgroups

T1D-CD individuals enrolled in the study were classified as CD_FIRST, CD_CONCOMITANT, and T1D_FIRST based on the timing of CD onset. We categorized T1D-CD individuals into: CD_CONCOMITANT if the onset of CD occurred within 4 months before or after T1D onset; T1D_FIRST if CD diagnosis was made > 4 months after the onset of T1D; CD_FIRST if CD diagnosis was made < 4 months before the onset of T1D.

### Data Collection

Data were collected retrospectively from medical records. Clinical, anthropometric, laboratory, and histological data were anonymously recorded in a database using a unique progressive identification code.

The following data were collected: age, gender, anthropometric characteristics (weight, height, body mass index (BMI), BMI Z-score), ethnicity, family history of CD and other autoimmune diseases (thyroiditis, psoriasis, rheumatoid arthritis, inflammatory bowel disease, vitiligo, etc), IgA and Anti-TTG IgA titers, anti-endomysial IgA antibodies, anti-GAD, anti-IA2, anti-IAA, anti-ZnT8, HbA1c, and diabetic ketoacidosis (DKA) at the onset of T1D, defined according to the definition of the ISPAD society (21).

Data regarding chronic or intermittent diarrhea, chronic abdominal pain, constipation, short stature/growth failure/weight loss, iron deficiency anemia/fatigue, recurrent vomiting, dermatitis herpetiformis-like rash, spontaneous fractures, convulsions, hypertransaminasemia, HLA-DQ2/DQ8 haplotypes, and histological evaluation (according to Marsh's classification) were collected at CD diagnosis.

Due to the retrospective design and involvement of multiple centers in the study using different anti-TTG IgA assay methods, we compared the ratio of the measured value of anti-TTG IgA to the ULN, rounded to whole numbers, and expressed as fold-anti-TTG IgA.

### Statistics

Continuous variables were screened for normal distribution using the Kolmogorov-Smirnov and Shapiro test. Anti-TTG IgA and HbA1c values were not normally distributed; therefore, a statistical analysis was performed using nonparametric tests. Categorical variables are presented as frequencies and percentages, while continuous variables are presented as mean ± SD. Differences between groups of continuous variables were analyzed with Student's *t* test for paired samples (for normally distributed variables) or with the Mann-Whitney test (for non-normally distributed variables). A chi-squared test with Fisher's test was used to evaluate differences in categorical data. ANOVA test, followed by Duncan post hoc analysis, was used for comparisons between groups for normally distributed variables; alternatively, the nonparametric Kruskal-Wallis test was used in case of variables not normally distributed. Spearman's correlations have been used to analyze the correlations between the different variables.

Receiver operating characteristic (ROC) curve analysis was used to determine the optimal anti-TTG IgA cutoff value for performing diagnostic biopsies for CD; Student *t* test was adopted to compare means, and Chi-square for proportions between 2 groups. Sensitivity, specificity, positive predictive values (PPV), and negative predictive values (NPV) were calculated. Sensitivity and specificity were tested by the McNemar test, and the predictive value by the weighted generalized score statistic. The significance of the test is fixed at *P* < .05.

## Results

### Case and Control Group Characteristics

Among 6933 cases of T1D that occurred between January 2010 and December 2019, 556 were diagnosed as T1D-CD (8%); of these, 95 were excluded due to incomplete data regarding the CD diagnosis. The missing data were randomly missing. Consequently, the study sample consisted of 461 patients (56% female, mean age 7.2 ± 4.0 years), recruited from the 23 centers. A group of 141 individuals with T1D only were concurrently enrolled as controls (52% female, mean age 7.6 ± 3.6 years). There was no difference according to age between the 2 groups (*t* = −0.9, *P* = .32). The baseline data and characteristics of the T1D-CD and T1D only individuals are described in Table 1.

**Table 1.** Baseline data and parameters of all cases (n = 461) and controls (n = 141)

	T1D-CD	T1D only	*P* value	CD_FIRST	CD_CONCOMITANT	T1D_FIRST
Number	461	141		25 (5.6%)	155 (33.4%)	281 (61%)
Age at T1D onset (mean ± SD)	7.2 ± 4.0 years	7.6 + 3.6 years	n.s.			
Male (%)	203 (44)	68 (48)	n.s.			
Caucasian ethnicity N (%)	409 (89.3)	139 (98.6)	n.s.			
BMI Z-score at T1D onset (mean ± SD)	−0.5 ± 1.08	0.88 ± 0.91	<.001	−0.57 ± 1.15 *P* value < .001	−0.56 ± 1.11 *P* value < .001	−0.52 ± 1.04 *P* value < .001
Personal history of other autoimmunity N (%)	116 (tot. 238) (50.4%)	34 (tot. 138) (24.4%)	<.001	13 (17)−76.4% χ2 19.46, *P* value < .001	37 (91)−40.6% χ2 6.76, *P* value = .03	70 (130)−53.8% χ2 24.46, *P* value < .001
Family history of autoimmunity N (%)	181 (tot. 415) (43.6%)	27 (tot. 140) (19.2%)	<.001	18 (tot. 25)−72% χ2 29.7, *P* value < .001	65 (142)−45.7% χ2 22.5, *P* value < .001	98 (248)−39.5% χ2 16.77, *P* value < .001
Fold-anti-TTG IgA (mean ± SD)	42.75 ± 93.14	0.98 ± 9.2	.001			
HbA1cCD mmol/mol (mean ± SD)	65.75 ± 22.39	61.34 ± 11.11	.08			

Abbreviations: BMI Z-score, body mass index Z-score; CD, celiac disease; CD_FIRST, celiac disease occurring before T1D onset; CD_CONCOMITANT, celiac disease occurring concomitantly with T1D onset; HbA1cCD, glycated hemoglobin at CD diagnosis; T1D, type 1 diabetes; T1D_FIRST, celiac disease occurring after T1D onset; TTG, transglutaminase.

The HbA1c values were available in all T1D_FIRST and in CD_CONCOMITANT individuals with T1D onset ≥ 0 months, whereas they were not collected in the individuals with CD_FIRST and in CD_CONCOMITANT with T1D onset < 0 months, according to international guidelines, which do not recommend HbA1c measurement in CD children without T1D.

**Figure 1.**
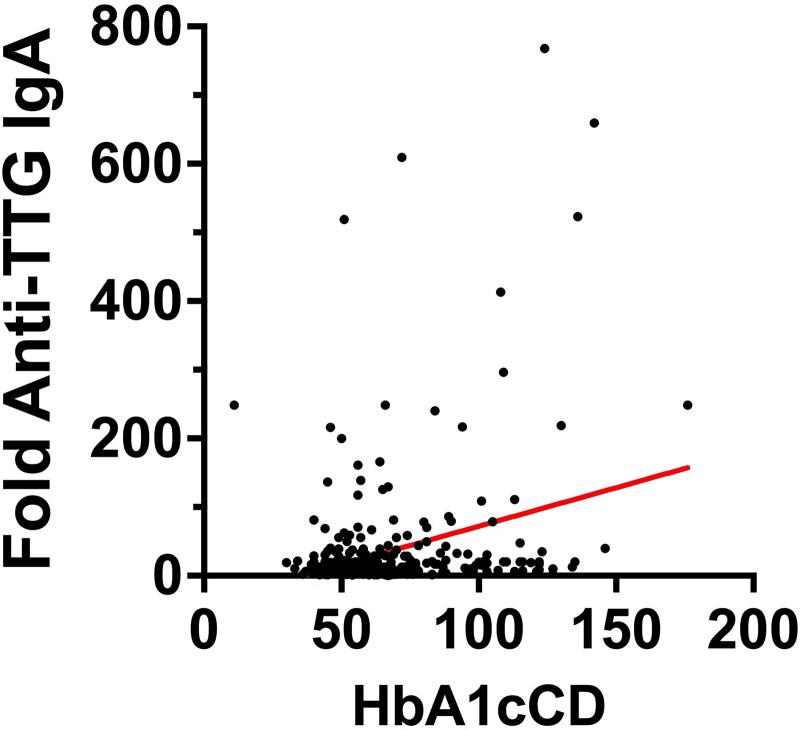
Linear relationship between log 10 (anti-TTG IgA) and HbA1c at CD diagnosis (HbA1cCD).

The fold-anti-TTG IgA at the CD onset in individuals with T1D (CD_CONCOMITANT and T1D_FIRST) significantly correlated with the level of HbA1cCD (Spearman *R* = 0.14, *P* = .0047) (Fig. 1). This association was also confirmed by dividing the individuals based on HbA1c targets of good and poor glucometabolic control according to American Diabetes Association Standards of Medical Care in Diabetes (22) (see data in Supplementary 1 (23)).

**Figure 2.**
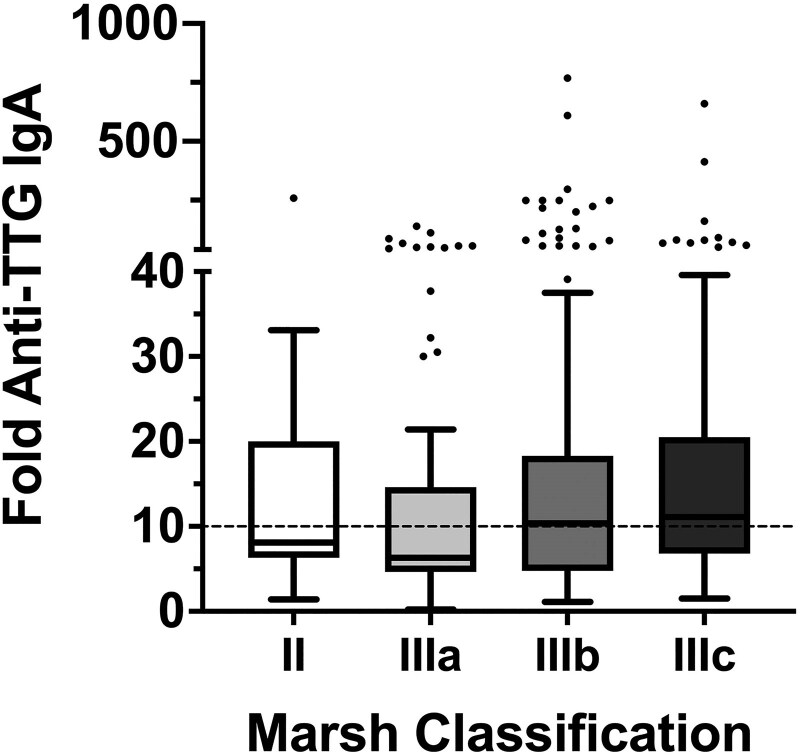
Distribution of log10(anti-TTG IgA) according to the different Marsh grades.

Analysis of fold-anti-TTG IgA and biopsy data at the CD diagnosis in the 336 children with T1D revealed that the mean of fold-anti-TTG IgA increase value was 33.1; the first quartile value was 5.9, while the third quartile was 18.5 (data not shown). Analysis of biopsy results (Marsh classification) revealed a different distribution of anti-TTG IgA according to the different Marsh grades (Fig. 2) and post hoc analysis unveiled significant differences within the 2 groups Marsh Stage 2 + 3A and Marsh 3B + 3C (*P* = .0048).

**Figure 3.**
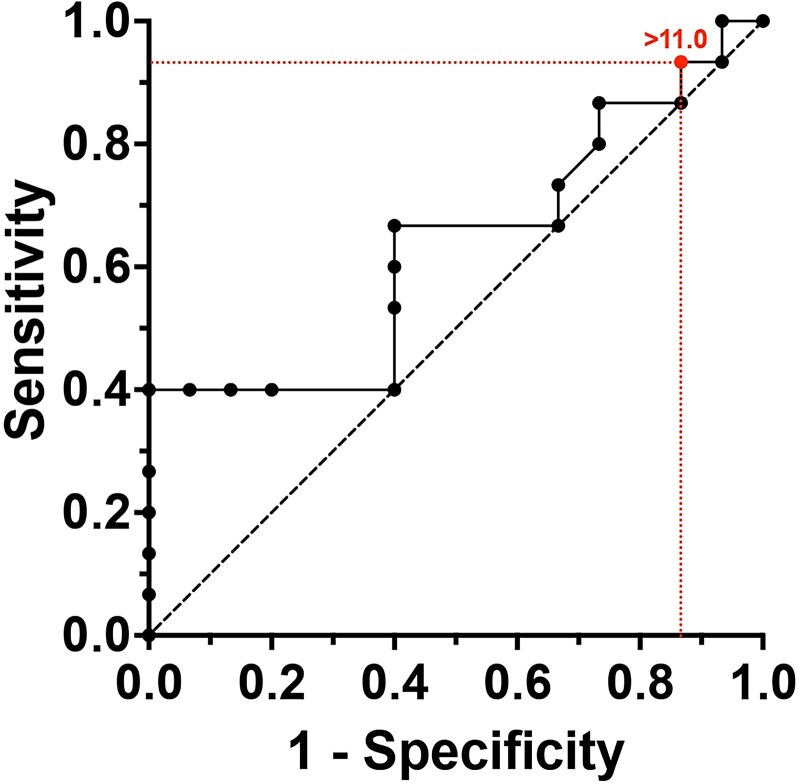
Receiver operating characteristic (ROC) curve.

Receiver operating characteristic (ROC) analyses were performed to determine the optimal anti-TTG IgA cutoff value for performing or not performing diagnostic biopsies for CD in individuals with T1D. The optimal cutoff level of anti-TTG IgA for detecting CD, in children with T1D, was 11 ULN (Fig. 3). This value was calculated on biopsy data and gave a sensitivity of 96% and a specificity of 14% for CD diagnosis, leading to a positive predictive value (PPV) of 45% and a negative predictive value (NPV) of 82% (Supplementary 2 (23)).

### Diabetes Autoantibodies Distribution

The autoantibodies assessed were anti-GAD, anti-IAA, anti-IA2, and anti-ZNT8. Data collected on the distribution of T1D autoantibodies, including numbers and percentages, are reported (Supplementary 3 (23)).

### Family History of Autoimmunity

Among T1D-CD individuals, 5.6% (25/461) were CD_FIRST, 33.4% (155/461) were CD_CONCOMITANT, and 61% (281/461) were T1D_FIRST (Table 1). Family history for autoimmunity was higher in T1D + CD (181/415 data available, 43.6%) than in T1D only individuals (27/140 data available, 19.2%) (χ^2^ 25.4, *P* < .001) (Table 1). In the CD_FIRST subgroup, the prevalence of family history of autoimmune disorders was 72% (χ^2^ 29.7 *P *< .001), in CD_CONCOMITANT was 45.7% (χ^2^ 22.5 *P *< .001), in T1D_FIRST was 39.5% (χ^2^ 16.77 *P* < .001) (Table 1).

The family distribution of autoimmune diseases among subgroups of T1D-CD and T1D only is described (Supplementary 4 (23)).

### Personal History of Autoimmunity

The T1D-CD individuals showed a greater burden of autoimmune disorders (116/238 data available, 50.4%) than T1D only (34/138 data available, 24.4%) (χ^2^ 10.02 *P* < .001) (Table 1). In the CD_FIRST, the prevalence of autoimmune disorders was 76.4% (χ^2^19.46 *P* < .001), in CONCOMITANT was 40.6% (χ^2^ 6.76 *P* = .03), in T1D_FIRST was 53.8% (χ^2^ 24.46 *P* < .001) (Table 1).

Prevalence of thyroiditis was approximately twice in the T1D-CD population (87/238 data available, 36.6%) than in controls (26/139 data available, 18.7%). Highest percentage of youths with autoimmune comorbidities other than thyroiditis was in the CD_FIRST (35.3%). After thyroiditis, the most common autoimmune disorder was psoriasis (26/238, 10.9%) (Supplementary 5 (23)).

### Other Clinical Features in Individuals With T1D-CD

A significant difference between T1D-CD and T1D-only, and between subgroups of T1D-CD and T1D only was observed for BMI Z-score at CD onset (*P* < .001 for all) (Table 1). Data regarding sex, ethnicity, biopsy results, and presence of DKA at T1D onset did not show any differences between T1D-CD and T1D-only individuals (Table 2).

**Table 2.** Other clinical features in individuals with T1D-CD

T1D-CD individuals	Data collected N (%)	Chi-squared	*P*
Sex	461 (100)	1.545	.462
Ethnicity/Caucasian	458/409 (99.3-89.3)	0.945	.624
Biopsy result/Marsh 3A + 3B + 3C	312/294 (67.7/94.2)	5.166	.523
Onset of T1D with DKA	418 (90.7)	3.494	.174

Abbreviations: CD, celiac disease; DKA, diabetic ketoacidosis; T1D, type 1 diabetes.

### Symptoms and Biopsy

Data on symptoms at CD diagnosis were available for 93.7% (432/461) of individuals with T1D-CD. Most of the cases were asymptomatic at onset (n = 303, 70%), while the most complained symptom at the onset of CD was chronic-recurrent abdominal pain (n = 43, 9.9%), followed by diarrhea, and short stature/failure to thrive/weight loss. Other symptoms, such as vomiting, dermatitis herpetiformis-like rash, spontaneous fracture, seizures, and hypertransaminasemia, were rarely reported (Supplementary 6 (23)).

CD diagnosis occurred in the first year after T1D onset in 69.4% of the cases (245/353). Only 19.54% of cases (69/353) were diagnosed with CD more than 5 years after T1D, with the latest diagnosis occurring approximately 13 years later (Supplementary 7 (23)).

## Discussion

The main finding of this study is that worse glucometabolic control is associated with both increased serum anti-TTG IgA titer and worse mucosal damage in children and adolescents with T1D at diagnosis of CD.

This association had not been previously reported and was also confirmed when the analysis was performed considering the 2 different groups of CD_CONCOMITANT and T1D_FIRST. Indeed, it is known that in CD, higher levels of anti-TTG are associated with more extensive mucosal damage, indicated by a Marsh grade of 2 or higher (13). The novelty of our study is that children with less extensive damage (2 + 3A) showed better glucometabolic control at CD diagnosis (reduced HbA1cCD) with respect to those with more extensive intestinal damage (3B + 3C).

This data was not expected, since HbA1c levels at CD diagnosis in individuals with T1D should be reduced due to nutrient malabsorption, and to the resulting increased risk of hypoglycemia (24, 25). However, our results are in line with a recent study on adults with T1D and newly diagnosed with CD, which shows an increased HbA1c compared to adults with T1D-only (7.5% vs 8.2%, *P* = .05) (26). Moreover, previous reports on populations with CD describe that individuals with active CD, and those on GFD with positive antibodies, showed a statistically significant correlation between levels of TTG IgA titers and serum levels of inflammatory cytokines (27). In a recent study, the amount of gluten was strongly correlated with an inflammatory profile in serum cytokines in infants who developed CD (28). A possible explanation for our result is that gastrointestinal inflammation and mucosal damage are triggered and maintained by dietary exposure to gluten in T1D individuals when CD occurs (29). Since inflammatory processes are usually involved in the progression of insulin resistance and associated with hyperglycemia in T1D (30), a higher anti-TTG IgA titer value and a worse histology could be explained by active CD, which in turn negatively influences the glucometabolic control.

In line with this, children with T1D-CD achieving anti-TTG antibody negativity with the GFD showed better HbA1c levels than those with anti-TTG antibody positivity (15, 16), the latter being associated with consistently higher HbA1c levels also in longitudinal analyses at 6 years (*P* < .001) (14, 15).

Further confirmation also comes from studies in which children with the double autoimmunity (T1D-CD) not adhering to the GFD, showed worse glucose sensor metrics as well as higher HbA1c ​​than those adhering to the GFD (18). Although other conflicting data show lower HbA1c in pediatric individuals with T1D-CD compared to T1D only, these studies lack evaluation of both adherence to the GFD and anti-TTG levels (31, 32).

The optimal anti-TTG IgA cutoff level at CD diagnosis in our large cohort confirms the same result reported by Wessels et al (12). The increase in the anti-TTG IgA cutoff from 10 ULN, evaluated for the general population, to 11 ULN should be considered in children with T1D to spare biopsy for CD diagnosis.

In the T1D-CD individuals, the prevalence of family history of CD and of personal history of other autoimmune comorbidities was higher compared to T1D-only individuals. Autoimmune thyroiditis was the most frequent comorbidity in T1D-CD individuals, as confirmed by the literature (7). The CD_FIRST subgroup showed a higher risk of developing additional autoimmune disorders, as confirmed in previous studies (33). To our knowledge, there are no studies demonstrating an immunomodulatory effect of gluten in terms of increased risk of autoimmunity in T1D, but this could be a possible hypothesis to be tested by further studies.

Considering T1D_FIRST individuals, the diagnosis of CD was most likely (84%) in the first 2 years. Despite this, CD may be diagnosed within the first 10 years of T1D history and, rarely, after 13 years (34). This evidence reinforces guidelines that currently suggest repeating anti-TTG within 2 years of T1D diagnosis and then again after 5 years.

Little is known about the screening with anti-TTG after 5 years from the onset of T1D (35). However, the same guidelines report that the anti-TTG measurement should be considered at other times in individuals with symptoms suggestive of CD.

Finally, our T1D-CD cohort was mostly asymptomatic (70%) at the time of CD diagnosis, as previously highlighted (36, 37), even though a significantly reduced BMI Z-score was observed in comparing T1D-CD to T1D-only individuals, thus confirming what was previously reported by Simmons et al (38).

The main strength of our study is the large number of children with T1D-CD enrolled, who had been diagnosed using a non-biopsy-sparing approach in the period prior to the new 2020 guidelines. This allowed us to evaluate the association between glucometabolic control, anti-TTG IgA titer, and mucosal damage at CD onset, as well as to demonstrate that a higher anti-TTG IgA cutoff for sparing biopsy could be necessary in performing CD diagnosis in youths with T1D. Furthermore, it emphasizes the importance of screening for T1D autoantibodies in CD individuals, which is not currently recommended. Limitations of this study include its retrospective design, the lack of a centralized laboratory, the lack of HbA1cCD in CD_FIRST, and the control group enrolled only at the coordinating center.

Implications for research include that gluten may have a role in glucometabolic control and prevention of cardiovascular complications in people with T1D-CD, and it is mandatory to obtain negativity of anti-TTG IgA as early as possible (15, 16, 18). Furthermore, although the data have been collected retrospectively, the higher risk of developing autoimmune comorbidities that subjects with CD_FIRST appear to have highlights that regular screening for other autoimmune comorbidities (eg, T1D) should be considered in the follow-up guidelines of subjects with CD.

## Data Availability

The datasets generated and analyzed during the present study are not publicly available but are available from the corresponding author on reasonable request.

## Abbreviations

CD: celiac disease
CD_FIRST: celiac disease occurring before T1D onset
CD_CONCOMITANT: celiac disease occurring concomitantly with T1D onset
DKA: diabetic ketoacidosis
ELISA: enzyme-linked immunosorbent assay
ESPGHAN: European Society of Pediatric Gastroenterology Hepatology and Nutrition
GAD: glutamic acid decarboxylase
GFD: gluten-free diet
HbA1c: glycated hemoglobin
HbA1cCD: glycated hemoglobin at CD diagnosis
IA2: insulinoma-associated protein 2
IAA: insulin autoantibodies
ISPAD: International Society for Pediatric and Adolescent Diabetes
ISPED: Italian Society for Pediatric Endocrinology and Diabetes
T1D: type 1 diabetes
T1D_FIRST: celiac disease occurring after T1D onset
TTG: transglutaminase
ULN: upper limit of normal
ZnT8: zinc transporter 8
